# Qualitative perspectives of the North Carolina healthy food small retailer program among customers in participating stores located in food deserts

**DOI:** 10.1186/s12889-021-11509-x

**Published:** 2021-07-27

**Authors:** Lindsey Haynes-Maslow, Stephanie B. Jilcott Pitts, Kathryn A. Boys, Jared T. McGuirt, Sheila Fleischhacker, Alice S. Ammerman, Nevin Johnson, Casey Kelley, Victoria E. Donadio, Ronny A. Bell, Melissa N. Laska

**Affiliations:** 1 Department of Agricultural & Human Sciences, NC State University, 512 Brickhaven Drive, Room 240c, Raleigh, NC 27695 USA; 2 Department of Public Health, East Carolina University, 115 Heart Drive, Room #2239, Greenville, NC 27834 USA; 3 Department of Agricultural and Resource Economics, North Carolina State University, 4306 Nelson Hall, Raleigh, NC 27695-8109 USA; 4 Department of Nutrition, University of North Carolina Greensboro, 319 Colllege Avenue, 318 Stone Building, Greensboro, NC 27412 USA; 5 Georgetown University, Law Center, Washington, DC USA; 6 Department of Nutrition, University of North Carolina at Chapel Hill, CB# 7426, 1700 MLK Jr. Blvd, Room 239, Chapel Hill, NC 27599-7426 USA; 7 University of North Carolina at Chapel Hill, School of Medicine, Division of Geriatric Medicine, Center for Aging and Health, 5003 Old Clinic CB#7550, Chapel Hill, NC 27599 USA; 8 Department of Social Sciences and Health Policy, Division of Public Health Sciences, Wake Forest School of Medicine, Medical Center Boulevard, Winston-Salem, NC 27157 USA; 9 Epidemiology & Community Health, University of Minnesota, 1300 South 2nd Street, WBOB Suite 300, Minneapolis, MN 55454-1015 USA

**Keywords:** Healthy corner store, Food policy, Nutrition legislation, Qualitative data collection and analysis, Customer perspectives

## Abstract

**Background:**

The North Carolina Healthy Food Small Retailer Program (NC HFSRP) was established through a policy passed by the state legislature to provide funding for small food retailers located in food deserts with the goal of increasing access to and sales of healthy foods and beverages among local residents. The purpose of this study was to qualitatively examine perceptions of the NC HFSRP among store customers.

**Methods:**

Qualitative interviews were conducted with 29 customers from five NC HFSRP stores in food deserts across eastern NC. Interview questions were related to shoppers’ food and beverage purchases at NC HFSRP stores, whether they had noticed any in-store efforts to promote healthier foods and beverages, their suggestions for promoting healthier foods and beverages, their familiarity with and support of the NC HFSRP, and how their shopping and consumption habits had changed since implementation of the NC HFSRP. A codebook was developed based on deductive (from the interview guide questions) and inductive (emerged from the data) codes and operational definitions. Verbatim transcripts were double-coded and a thematic analysis was conducted based on code frequency, and depth of participant responses for each code.

**Results:**

Although very few participants were aware of the NC HFSRP legislation, they recognized changes within the store. Customers noted that the provision of healthier foods and beverages in the store had encouraged them to make healthier purchase and consumption choices. When a description of the NC HFSRP was provided to them, all participants were supportive of the state-funded program. Participants discussed program benefits including improving food access in low-income and/or rural areas and making healthy choices easier for youth and for those most at risk of diet-related chronic diseases.

**Conclusions:**

Findings can inform future healthy corner store initiatives in terms of framing a rationale for funding or policies by focusing on increased food access among vulnerable populations.

## Introduction

Adequate consumption of fruits and vegetables, whole grains, healthy oils, and lean protein is essential for optimal health throughout the lifespan [1]. However, the ability to maintain healthy eating patterns is contingent upon availability, affordability, and acceptability of healthy foods and beverages [2–5]. Maintaining healthy eating patterns is even more difficult for rural and low-income populations, which may contribute to the higher rates of diet-related chronic diseases observed in these communities [6–8]. Studies indicate such residents face significant barriers when attempting to purchase healthy foods and beverages, including economic barriers, lack of adequate transportation, and residence in a food desert (too few healthy food venues) or a food swamp (too many venues offering less healthy foods and beverages) are commonly cited barriers [9, 10].

One of the reasons for this limited access to healthy food is that there is a high prevalence of small food stores that do not stock healthier food options [9–14]. Residents of these communities, particularly those with limited transportation, often rely on these stores as a major source of food [9, 12–14]. Generally, these stores do not offer a wide variety of healthier foods and beverages [11]. To improve access to healthy foods and beverages in rural and underserved areas, healthy corner store policies have been implemented across a number of jurisdictions [15, 16]. Indeed, several states have passed legislation designed to facilitate the sale of healthy foods and beverages through small food retailers to increase access to healthy foods in areas where there is limited access [17]. These legislative efforts vary considerably in their scope, the mechanisms used to incentivize store participation, and the agencies assigned to implement these programs.

To date, research on healthy corner store policies suggests they generally improve customers’ intent to consume healthier foods and beverages [16, 18] and increase availability of healthier foods and beverages [15, 19, 20]. However, mixed quantitative findings regarding whether healthy corner store policies improve the nutritional quality of customers’ diets have hindered our understanding of their impacts [20–24]. Qualitative data may help elucidate a deeper understanding of influential factors not fully captured by quantitative measurement. Therefore, the purpose of this study was to qualitatively examine customer perceptions among regular shoppers at stores participating in the North Carolina Healthy Food Small Retailer Program (NC HFSRP) [25]. We sought to understand regular customers’ views of the healthier selections, if they supported the NC HFSRP, and if their food and beverage choices had been impacted by the healthier options available through the NC HFSRP.

## Methods

### The North Carolina healthy food small retailer program

The NC HFSRP was established to provide funding for small food retailers located in United States Department of Agriculture (USDA)-defined food deserts to increase stocking of healthy foods and beverages with the hope of improving the eating patterns of local residents and benefiting small food retailers [25]. According to the USDA, census tracts are considered a food desert if they are both low-income and have low food access [26]. Low-income census tracts are those with poverty rates equal to or greater than 20% or a median household income that does not exceed 80% of the state’s median household income (rural/urban) or urban area median household income. Low food access is defined as at 33% of the population living more than 1 mile (urban) or 10 miles (rural) from the nearest supermarket or large grocery store [26]. Beginning in 2016 and through 2018, the NC Legislature appropriated $250,000 annually to fund the NC HFSRP. The NC Department of Agriculture and Consumer Services served as the implementing agency. This program was available for small food retailers (with less than 3000 heated square feet of retail space) who were willing to (or already did) accept Supplemental Nutrition Assistance Program (SNAP) and the Special Supplemental Nutrition Program for Women, Infants, and Children (WIC) benefits.

Small food retailers were eligible to apply for grants up to $25,000. These funds could be used to purchase and install equipment necessary for stocking nutrient-dense foods and beverages, including fresh vegetables and fruits, whole grains, nuts, seeds, beans and legumes, low-fat dairy products, lean meats, and seafood [27]. In this qualitative study, we examined perceptions of the NC HFSRP overall, perceived changes in the food store environment of participating stores, and changes in customers’ purchasing and/or consumption habits. This data collection effort was part of a larger mixed-methods independent evaluation of the impact of the NC HFSRP on the store food environment as well as purchasing and consumption habits among customers [28]. This study includes three of the six stores from the first year of funding, and two of the five stores from the second year of funding [27].

### Study setting and participants

In-depth, semi-structured interviews were conducted with 29 customers from five NC HFSRP stores. Selection of HFSRP stores occurred on a rolling basis through an application process [27]. Stores voluntarily applied to be in the HFSRP. For this study, the qualitative customer interviews took place in HFSRP stores located in five eastern North Carolina counties (Bertie, Halifax, Onslow, Pasquotank, and Edgecombe). See Table 1. Customers were approached on weekdays, during normal business hours, and asked if they were interested in completing a 10–15 minute interview. In each of the five stores, between 3 to 10 participants were recruited and consented to being interviewed. Participant eligibility criteria included: being a self-defined ‘regular’ customer of the store, at least 18 years of age, and able to speak and understand English. Interviews were conducted by the study’s principal investigator or trained research assistants. Interviews were digitally recorded and notes were taken on paper, and then interviews were transcribed verbatim using an online transcription service. Participants were compensated for their time with a $10 Walmart gift card upon interview completion. The study was reviewed and approved by the East Carolina University Institutional Review Board [UMCIRB 16–002420]. All participants signed an informed consent form.

**Table 1:** County characteristics for the five counties where Healthy Food Small Retailer Program (HFSRP) stores were located, in which customers were interviewed

County	Percent Poverty	Percent Food Insecurity	Percent obese adults	Distance from store in that county to closest grocery store (miles)	Percent with a bachelor’s degree or higher (ages 25 and older)
Bertie	24.2%	22.0%	43.0%	13.7	13.6%
Edgecombe	21.0%	24.0%	38.0%	1.07	13.6%
Halifax	23.8%	24.0%	44.0%	15.3	14.4%
Onslow	12.5%	15.0%	29.0%	5.41	22.9%
Pasquotank	14.3%	19.0%	39.0%	1.83	22.1%
**State**					
North Carolina	13.6%	13.9%	34.0%	–	31.3%

Sources

https://www.census.gov/quickfacts/fact/table/pasquotankcountynorthcarolina,onslowcountynorthcarolina,halifaxcountynorthcarolina,edgecombecountynorthcarolina,bertiecountynorthcarolina,NC/PST045219

https://www.countyhealthrankings.org/app/north-carolina/2020/rankings/bertie/county/outcomes/overall/snapshot

https://www.countyhealthrankings.org/app/north-carolina/2020/rankings/pasquotank/county/outcomes/overall/snapshot

https://www.countyhealthrankings.org/app/north-carolina/2020/rankings/halifax/county/outcomes/overall/snapshot

https://www.countyhealthrankings.org/app/north-carolina/2020/rankings/onslow/county/outcomes/overall/snapshot

https://www.countyhealthrankings.org/app/north-carolina/2020/rankings/onslow/county/outcomes/overall/snapshot

https://www.countyhealthrankings.org/app/north-carolina/2020/measure/factors/139/data

https://www.census.gov/quickfacts/fact/table/NC/PST045219

https://stateofchildhoodobesity.org/adult-obesity/

https://www.americashealthrankings.org/explore/health-of-women-and-children/measure/food_insecurity_household/state/NC

### Semi-structured interview guide

Data were collected using semi-structured interviews to learn more about customers’ perspectives of the NC HFSRP. An interview guide was developed through several meetings of the research team. Questions focused on shoppers’ food and beverage purchases at NC HFSRP stores (“Have your food or beverage purchasing habits at this store changed since the program began?”; “Has the overall amount of healthy food or beverage items you buy changed since this store started carrying healthier foods?”; and, “Has the overall amount of healthy food or beverage items you consume changed since the store starting carrying healthier foods?”); whether they had noticed any in-store efforts to promote healthier foods and beverages, their suggestions for promoting healthier foods and beverages, their familiarity with and support of the NC HFSRP, and how their shopping and consumption habits had changed since implementation of the NC HFSRP. Participants were also asked to provide basic demographic information including gender, age, race, ethnicity, educational attainment, and household income.

### Data analysis

First, three transcripts were independently reviewed by the principal investigator, study coordinator, and two graduate research assistants. Each member of the research team prepared an independent codebook consisting of codes, operational definitions, and illustrative quotes. Codes included those derived from the interview guide (deductive codes) and those that emerged from the interview data (inductive codes). The research team then discussed their independently-developed codebooks to create a consensus codebook which was subsequently used to code all transcripts. Each transcript was double-coded (separately coded by each of two research team members), and these codes were reconciled in a series of consensus-building meetings between team members. Reconciled and coded transcripts were uploaded into NVivo (Version 12, QSR International, Melbourne, Australia), and reports were generated for each code. A thematic analysis was conducted based on code frequency, and depth of participant responses for each code [29].

## Results

A summary of participant self-reported demographic characteristics is presented in Table 2. Among the 29 participants, the majority were female (59%) and the majority were between 18 and 64 years of age. Approximately 55% were Black/African American, 41% White, and 3% American Indian/Alaska Native. Nearly 45% had a high school degree or less. Half of the participants (52%) were employed full-time.

**Table 2:** Participant Demographics among Customers of Stores in the North Carolina Healthy Food Small Retailer Program (*N* = 29)

Characteristic	Frequency	Percent
**Gender**		
Male	12	41
Female	17	59
Other	0	0
Prefer not to answer	0	0
**Age**		
18–29	6	21
30–49	7	24
50–64	10	35
65+	4	14
Prefer not to answer	2	7
**Race/ethnicity**		
American Indian or Alaskan Native	1	3
Asian	0	0
Black or African American	16	55
Native Hawaiian or Pacific Islander	0	0
Hispanic or Latino	0	0
White	12	41
Other	0	0
Prefer not to answer	0	0
Education attainment		
Less than high school	0	0
Some high school	3	10
High School/GED	10	35
Associate/Technical Degree	4	14
Some College or More	12	41
Annual household income		
Less than $25,000	6	21
$25,000 to $50,000	10	35
$50,000 to $75,000	1	3
$75,000+	6	21
Don’t know	3	10
Prefer not to answer	3	10
Employment status		
Full-time	15	52
Part-time	6	21
Unemployed	5	17
Retired	3	10

Through coding, six main themes emerged from the interviews based on frequency of the code and depth of discussion. These themes included the following, in order of frequency of codes: 1) Change in food purchasing behavior; 2) Stocking suggestions for additional healthy food items; 3) Awareness of current healthy food promotion and advertising efforts; 4) Suggestions for how to promote healthy foods and beverages in the future; 5) Opinions on the NC HFSRP; and, 6) Opinions concerning NC HFSRP funding. Table 3 describes each theme and its operational description, as well as the number of interviews in which each theme is referenced, and the total number of references for each theme.

**Table 3:** Code, Operational Definitions, and Code Frequency for Qualitative Analysis of data collected among Customers of North Carolina Healthy Food Small Retailer Program Stores (N = 29)

Code	Operational Definitions	Number of Interviews where the code Referenced	Total Number of References for the Code	Illustrative Quote
Change in Food Purchasing Behavior	Participant describes changes in their purchasing and consumption of healthier foods based on the HFSRP	29	123	“We buy more fish. We almost never buy meat. We spent a lot of time in the produce section.” [Edgecombe County, Male] “Instead of getting her [daughter] a piece of pizza, I bought her two bananas” [Bertie County, Female]
Stocking Suggestions for Additional Healthy Food Items	Participant describes items they would like to see in the store which are not currently in the store.	28	43	“If they had grilled fish instead of fried fish, I would absolutely buy that instead.” [Bertie County, Female]
Opinions on the NC HFSRP	Participant describes what they think about the HFSRP	29	37	“I think it’s wonderful that they [the store] can get something out of it for all they give back.” [Edgecombe County, Male]
Awareness of Current Healthy Food Promotion and Advertising Efforts	Participant describes health promotion/ nutrition promotion efforts of the store owner	29	31	“They’ve added these fruits and veggies, and they have some really cool alternative drinks to soda.” [Edgecombe County, Male]
Suggestions for How to Promote Healthy Foods and Beverages in the Future	Participant describes other strategies used to make healthier choices easier for customers to make.	29	30	“I think they could use social media to a stronger degree.” [Edgecombe County, Male]
Opinions Concerning NC HFSRP Funding	Participant response to whether or not the HFSRP should continue to be funded	28	28	“I think it sounds like a great program to fund.” [Edgecombe County, Female]

### Change in food purchasing behavior

Table 4 includes questions, responses, and illustrative quotes regarding NC HFSRP-related purchase and consumption changes. This theme was most frequently discussed by participants. When asked if their food and beverage purchasing behaviors at the store had changed since the NC HFSRP, 15 (52%) said “yes” they had increased healthy food purchasing. Most participants indicated they now purchased more fruits, vegetables, and whole grains than before NC HFSRP implementation. One participant even commented on a store’s locally made granola, “*I do have a 15 year-old son. I would say buying this granola instead of store granola, I think this is better. And it’s trickled down to my husband and my son, and they truly like it.*” [White Female, Edgecombe County]. Another commented, “*usually I have to take a 30-minute ride to buy an apple. Now I can ride five minutes up the road and buy one.*” [White Male, Halifax County]. Importantly, having the convenience of purchasing healthier items appeared to be connected with increased purchases. Overall, participants reported that having healthier items at the corner store closer to their home saved them from having to drive further distances to grocery stores, supermarkets, or supercenters to get fresh produce or a variety of healthy food items.

**Table 4:** Changes in Food Purchasing and Consumption Behavior among Customers in North Carolina Healthy Food Small Retailer Program Stores (n = 29 customers in 5 stores)

Change in Food Purchasing and Consumption Behavior	Responses and (%) Responding^**a**^	Illustrative quote
Have your food or beverage purchasing habits at this store changed since the program began?	Increased purchase of healthy foods at the store: 15/29, 52% No change in purchases at the store: 13/29, 45%	*Interviewer*: Overall, has your food or beverage purchasing habits at this store changed since the program began? *Respondent*: They actually have … I hadn’t seen them in a while like the tangerines in the cup and … I’ve actually ate more tangerines and fruits in the cup and stuff because they’re there now. [Pasquotank County, Male]
Has the overall amount of healthy foods or beverage items you **buy** changed since the store started carrying healthier foods?	Yes, increased purchase of healthy foods: 14/29, 48% No, healthy food purchases have stayed the same: 13/29, 44%	*Interviewer*: Has the overall amount of healthy foods or beverage items you buy changed since the store started carrying healthier foods? *Respondent*: I do buy more bananas because they’re right there. [Bertie, Female]
Has the overall amount of healthy food or beverage items you **consume** changed since the store started carrying healthier foods?	Purchasing healthy food in addition to regular purchases: 12/29, 41% Purchasing healthier food instead of less healthy food 8/29, 28% Unsure about consumption changes: 7/29, 14%	*Interviewer*: When you purchase these healthier items, are you picking these items in addition to items that you typically buy or are you buying them instead of less healthy options? *Respondent*: In addition. Yeah. I think this is one of the few places in Tarboro at least that gives you the healthier options. [Edgecombe County, Male]

^a^Notes: In instances where the percent of respondents does not sum to 100, the remaining percent were not asked the question or did not respond

When asked if the overall amount of healthy food or beverage items they purchased from the store changed since the NC HFSRP, 15 (52%) participants reported that the amount had increased, 10 (34%) stated it stayed the same, and 4 (14%) had no response or were not asked the question. A participant remarked that now they put more thought into their food and beverage purchases: “*… I used to be just a straight junk food kid, but now I try to balance it out*.” [Black/African American Male, Onslow County]. Many participants discussed purchasing healthier foods and beverages from the store for family members, friends, and even co-workers. For example, when asked if her purchasing habits had changed, one mother said, “*I noticed today when I came in that they [the store] had bananas on the rack, so we ended up grabbing one of those. My daughter loves fruits so if those are available at a gas station, we buy those*.” [White Female, Bertie County]. Others noticed the healthier options in the store but had opted not to buy them.

In terms of food or beverage consumption changes since the NC HFSRP, participants were asked if the healthy food items they purchased in the store were in addition to the items they would normally buy or if they were purchasing healthier food and beverages instead of less healthy food options. Among the 27 participants who responded to this question, 12 (41%) indicated that they were purchasing healthy food in addition to their regular purchases (healthy food complements to existing purchases). An additional eight respondents (28%) indicated that they were purchasing healthier food instead of less healthy food (substituting healthier foods for less healthy food), and seven (24%) were not sure of the impact the healthy foods on their shopping habits. One participant responded he bought healthier food in addition to his regular store purchases, stating: “*This is one of the few places in [town name] that at least gives you the healthier options.*” [White Male, Edgecombe County]. One caregiver commented that she was buying healthy food in lieu of unhealthy food for her daughter: “*Instead of getting her a piece of pizza, I bought her two bananas. She loves bananas*.” [White Female, Bertie County].

### Stocking suggestions for additional healthy food items

Across the interviews, participants offered 43 suggestions concerning what healthy food or beverage items they would like to see added to their store’s inventory. Fruits were the most popular response (*n* = 16); specific suggestions included fruit cocktail, apples, oranges, grapes, and plums. Other suggestions included vegetables (*n* = 3) and pre-made salads (n = 3).

### Awareness of current healthy food promotion and advertising efforts

When asked if participants noticed any changes to the ways the store promoted healthier food and beverages over the past few months, 17 of 29 participants said “yes.” When asked what they had noticed, most noted increased quantity and variety of produce, signs and other promotional materials related to healthy foods, and new equipment. As one participant said, “*They’ve added these fruits and veggies, and they have really cool alternative drinks to soda*.” [White Male, Edgecombe County]. Another participant noticed the store started accepting federal food assistance benefits, the Supplemental Nutrition Assistance Program (SNAP), formerly referred to as “food stamps”: “*They even do the EBT (SNAP Electronic Benefit Transfer). They do that, which I think is really nice, because a lot of the organic kind of places aren’t really geared toward that. And … we’re a very poor community, and I think it’s awesome that she [the store owner] does this.*” [White Female, Edgecombe County]. Some participants recalled seeing free recipe cards associated with healthy food items sold at the store, as well as promotional signs: “*Advertising’s always the key to anything and what she [the store owner] started doing now with the signs, that makes a big difference. People have to know what you have to be able to come get it*.” [Black/African American Male, Pasquotank County].

### Suggestions for how to promote healthy foods and beverages in the future

Multiple suggestions were offered regarding additional approaches stores could use to promote healthy items. Many people suggested advertising via social media and at community functions. As one participant said, “*… you just have to advertise that a little bit more so people know exactly what’s in here …*.” [Black/African American Female, Pasquotank County]. Another participant suggested more outdoor advertising so people driving by could see what was available in the store. Additionally, several participants discussed wanting more nutrition information regarding specific food and beverages: “*Put the little signs that it breaks down the calories and the nutrients, or what fruit has vitamin C in it or what had vitamin D in it. Just have one of those charts up*.” [Black/African American Male, Onslow County].

### Opinions on the NC healthy food small retailer program

While participants recognized healthy changes in the stores, among the 29 participants, only three were aware of the NC HFSRP. After explaining the premise of the program, all 29 participants agreed it was a program that could provide benefits to the community. In addition, several participants noted the program could benefit youth in their (rural) communities since they often purchase items from these stores. As one participant commented:

*“I think it’s good because you got kids, little kids that come here. You know and they want stuff. And before it was like just a lot of junk food, but now, you’ve got healthy stuff like vegetables and fruits that are constantly being changed out now. This is perfect because my friend, she lives across the road. She’s got kids and they always say, ‘I want junk food.’ But now I can go, ‘Hey, instead of junk food, how about an apple?’”* [White Male, Pasquotank County].

Multiple participants liked the goal of improving healthy options in their rural area. One woman who said the program was “*awesome*” went on to say, “*In these types of neighborhoods, if you put a store with fresh fruits and have different variety … like a lot of people don’t have a car.”* [Black/African American Female, Pasquotank County]. Another commented that for rural communities, this program is important for healthy food access: “… *because I like to eat the vegetables and have fruit. Then I won’t have to go all the way to town. I can just come right here and get it.”* [Black/African American Female, Bertie County]. Lastly, many participants discussed health issues. When one participant was asked what influenced her to buy healthy food/beverages, she said, “*That everybody in my family got high blood pressure … My mom had it. My dad. I got three brothers living. I got a son. Two nephews. Yes. It’s there*.” [Black/African American Female, Bertie County]. Participants noted the healthier options facilitated a healthier lifestyle that was important to prevent or ameliorate diet-related chronic diseases.

### Opinions concerning NC HFSRP funding

While most participants had never heard of the NC HFSRP, when asked if the NC Legislature should continue funding it, all responded “yes”. Some explained their reasoning by stating it helped increase rural food access and it was good for health: “*I hope they will [fund it]. It would help us a lot. Living out here in the county, you can’t always go to the store. Having stuff like this is helping us and everybody else too.*” [Black/African American Female, Halifax County]. Another commented, “*I feel like if that’s going to be a program that’s going to help people, especially when it comes to health and then trying to actually put food in someone’s home, then yeah, I feel like they should definitely fund that*.” [Black/African American Male, Onslow County]. Another participant focused on program efficacy, stating, “*If it’s working or if it’s profitable, you know, doing what it’s supposed to do, and the funds are actually going where they should go. Yeah [the legislature should fund it]*.” [White Male, Onslow County].

## Discussion

This study’s findings provide a deeper understanding of the impact of the NC HFSRP program from the perspective of customers. Although few participants knew about the NC HFSRP, they were aware of changes within the store and some customers reported that the changes encouraged increased purchasing of healthier foods and beverages. When a description of the NC HFSRP was provided to study participants, all approved of the state funded program’s purpose. Participants discussed the benefits of the program, including increased healthy food access in their rural and underserved communities, which they felt was especially important for youth and individuals with diet-related chronic diseases. There was also an overall agreement that being able to purchase healthier food and beverages closer to home – instead of driving long distances to larger stores – was helpful. Participants provided suggestions for further improvements to store offerings, including more pre-cut and fresh fruit options. Lastly, participants discussed the importance of marketing and advertising healthier foods and beverages in the stores.

There were mixed responses in terms of the NC HFSRP changing customers’ shopping habits regarding the amount of healthy food and beverage items purchased and increases in healthy food and beverage consumption. Overall, approximately half of participants reported making healthier food and beverage changes either through changing their overall shopping habits, changing amounts of healthy foods/beverages purchased, and/or types/amounts consumed. Other studies have found limited evidence of healthy corner store conversions and healthy food policies impacting dietary behaviors among customers [30–32]. For example, in two California food swamps, researchers found that among three corner stores that made in-store changes to promote the purchase and consumption of healthy foods and beverages, no changes were found among customers [30]. However, there were improvements in perceived healthy food access and more positive perceptions of corner stores [30]. Another study that evaluated the impact of the Minneapolis 2015 Staple Food Ordinance, which mandated minimum healthy food and beverage stocking requirements in small stores, found there was little impact on healthy food purchases by customers that frequented these stores [24]. However, it is important to recognize that shifting eating patterns is not an easy, nor quick, process, often taking both individual-level and policy and environmental-level changes to support healthier food and beverage choices [31, 32]. It should also be noted that among small retail food outlets represent a relatively small amount of overall food and beverage purchasing, compared to other retail food outlets [33]. In a systematic review of 64 studies focused on improving healthy food consumption and purchases in the retail environment, Karpyn and colleagues found that 56 studies showed at least one positive effect [32].

One limitation with the NC HFSRP’s implementation was that, even in the areas targeted by the program, relatively few customers in participating stores were aware of the program. This was frequently true even in cases where respondents had noticed changes in the store since its inception. As state funding for programs can be contingent upon perceived success and satisfaction among taxpayers, this is an important observation. While all participants in this study agreed that the NC Legislature should continue funding the program, going forward, it would be helpful to communicate more explicitly that the store changes are supported by the NC HFSRP. While the NC HFSRP did not include funding for advertising or branding, some stores partnered with local health departments, nutrition education programs such as SNAP-Education, or other local organizations to help purchase some marketing materials. This supports the need for both individual (educationally- and knowledge-based) as well as structural (policy and environmental change) strategies for promoting healthy eating among underserved populations [34].

Given the political importance of legislators being recognized for positive policies implemented in the community, and the potential benefit to sustaining such policies, including funds in the legislation for marketing and branding is important. In the case of the NC HFSRP, funding was limited to equipment purchases only. Additional funding for technical assistance and marketing could potentially have increased healthy food purchasing, as well as policy awareness, thus strengthening the case for this legislation.

This study found that many participants valued the state funded program because it increased overall convenient access to healthier food and beverages in rural communities. In addition, participants recognized that children shop at these stores without adult supervision; this program thus can foster opportunities for children to be exposed to healthier food and beverage choices. Participants also noted their shopping habits may have changed for the better due to desires to improve their health due to diet-related chronic diseases. When passing legislation, political battles are usually won based on framing arguments that resonate with public opinion and political will [35]. Therefore, in terms of future healthy corner store state-wide policies, stakeholders could frame arguments for the policy around healthy food access, improving children’s dietary choices and health and providing those with health problems alternative, healthier choices [36, 37]. In terms of political will, the HFSRP bill was co-sponsored by both Democrat and Republican legislators. It also passed with overwhelming bi-partisan support, likely due to the emphasis on supporting small businesses in rural communities as well as providing healthy food for residents of food deserts [25].

This study has several strengths. As one of the first studies to examine customer’s perceptions of a statewide policy to improve healthy purchases at small stores in rural and low-income areas, these findings offer insights and fill important gaps in the literature. The customer participants were racially/ethnically diverse and, through a qualitative approach, provided rich perspectives on the program’s impact on purchasing decisions.

While this research is novel in its approach, the study scope and setting do introduce some limitations. First, it is limited in geographic scope (five counties in one state) and sample size (29 customers) and therefore its findings have limited generalizability. Because customers were interviewed at the store, their responses could have been biased to be more socially desirable than they may otherwise have been due to the presence of store personnel. While one store’s NC HFSRP contract was pending at the time of the interviews, this store had not yet received the equipment for the program at the time of the interviews. However, the retailer had received similar equipment from another program. Lastly, some of the participants that agreed to be interviewed may have been more health conscious than others.

## Conclusions

Given the increasing number of healthy corner store policies, [17] the results of this study will be useful to both offer grounded insights into potential impacts of resource allocations towards environmental supports that promote healthy eating. Additionally, this study could help inform an approach other jurisdictions could adopt to evaluate their own programs. Our findings can inform future healthy corner store initiatives in terms of framing the rationale for funding – including improving food access in low-income areas, and empowering youth and those with diet-related chronic diseases to select healthier foods and beverages when in smaller food retail stores. More research is needed to determine the cost/benefit ratio of various state investments in promoting healthy eating among rural communities, as well as determine the level of support retail stores need to stock and promote healthier foods and beverages in a sustainable way. Future policies of this type could purposively include language and funding related to marketing as well as partnering with local health departments, SNAP-Education programs, or cooperative extension offices to promote purchase and consumption of the healthier options, ultimately to increase demand, financial sustainability, and overall health of rural and underserved communities.

## Data Availability

The (deidentified) datasets used and/or analysed during the current study are available from the corresponding author on reasonable request.
